# The isolation of the antagonistic strain *Bacillus australimaris* CQ07 and the exploration of the pathogenic inhibition mechanism of *Magnaporthe oryzae*

**DOI:** 10.1371/journal.pone.0220410

**Published:** 2019-08-12

**Authors:** Wenqian Chen, Lu Zhao, Hui Li, Yilun Dong, Hong Xu, Ying Guan, Songhao Rong, Xiaoling Gao, Rongjun Chen, Lihua Li, Zhengjun Xu

**Affiliations:** Rice Research Institute of Sichuan Agricultural University, Crop Ecophysiology and Cultivation Key Laboratory of Sichuan Province, Chengdu, China; Fujian Agriculture and Forestry University, CHINA

## Abstract

Biological control as a promising method to combat plant disease has gained public attention in recent years. In the present study, we isolated 12 strains resistant to *Magnaporthe oryzae* from western Sichuan subalpine soil. Among them, CQ07 exhibited remarkable activity against *M*. *oryzae*. The result of 16S rRNA sequence analysis revealed that CQ07 is approximately 99% similar to *Bacillus australimaris*. The sterilized culture filtrate of CQ07 inhibited the growth of *M*. *oryzae*, which motivated us to deduce the influence of CQ07 on the pathogenicity of *M*. *oryzae*. As shown by experimentation, sterilized culture filtrate (10 μl/ml) of CQ07 can delay and even suppress the germination of conidia and prevent the formation of appressorium in vitro and in vivo. In addition, by simulative field tests, the spraying of conidia suspension diluted with sterilized culture filtrate of CQ07 reduced infection of rice blast. To better control rice blasts, understanding the infection mechanism of *M*. *oryzae* and inhibiting the mechanism of the antagonistic strain is of great importance.

## Introduction

Rice blast caused by the filamentous ascomycete *Magnaporthe oryzae* is one of the most destructive diseases of rice around the world[1]. Outbreaks of rice blast disease are a serious and recurrent problem in China and other rice-growing regions[2]. Therefore, many people use antifungal drugs and plant disease-resistant cultivars to reduce the loss caused by rice blasts. On the one hand, chemical antifungal drugs have many advantages, such as high efficiency and inexpensive use. On the other hand, the overuse of chemical fungicides not only pollutes the environment but also makes pathogenic fungi increasingly drug resistant. Although the introduction of resistant cultivars can prevent disease efficiently, new variations may lead to the appearance of new pathogenic bacteria[3].

With increasing focus on sustainable development in agriculture, biological control as an environmentally friendly and promising strategy has emerged to the public[4]. In recent studies, there has been an increasing interest in the exploitation of rice blasts for biological control by using plant beneficial microorganisms, owing to their low toxicity and a lack of pathogen resistance[5]. At present, Bacillus have been found to be considerable biological control candidates in agriculture, with their high production of antifungal substances and resistance to extreme conditions. Earlier studies indicated that Bacillus can increase plant resistance to pathogenic fungi by colonizing plants or spraying a powder made from Bacillus on a plant, which exerts a biological control effect. In addition, Bacillus can not only produce antifungal substances but also promote the growth and increase the yield of plants[6–8]. Therefore, the potential value of Bacillus in biological control is immeasurable.

Many studies are currently devoted to isolating antifungal components from Bacillus or colonizing plants with Bacillus to control rice blast[9–11], but little research has been performed on inhibiting pathogenic mechanisms. *M*. *oryzae* is a model organism for molecular biology investigations and studying the pathogenesis of filamentous fungi. To control rice blast disease effectively, understanding the infection process and mechanisms of pathogenicity are important premises. The process of infecting a rice host mainly includes contact, invasion and expansion. The spread of rice blasts is often caused by the dissemination of *M*. *oryzae* conidia through the air. Conidia attach to the leaf surface, and then, a dome-shaped cell with a complex cell wall structure, an appressorium, contacted by the cuticle is formed at the ends of germ tubes, which can penetrate directly through the host cuticle by its enormous turgor pressure[12, 13]. Observation of the formation process of the infection structure and real-time monitoring of the dynamic growth of rice blasts in host rice tissue will help reveal the interaction process between *M*. *oryzae* and rice, which is of great significance for the biological control of rice blasts. Green fluorescent protein (GFP) has been widely used as a labeling tool in life science research, so the GFP-labeled *M*. *oryzae* strain Guy11 was used for the following tests.

A strain of bacteria displaying strong suppression against *M*. *oryzae* was selected from western Sichuan subalpine soil and named CQ07. Through the examination of the morphological, biochemical and physiological properties and 16S rRNA sequence analysis, the strain was identified. Our study observed germination of conidia, formation of an appressorium, growth of mycelium, cellular permeability in vitro and infection process in plant tissues of *M*. *oryzae*. Furthermore, inhibition tests of sterilized CQ07 culture filtrate on infection structure development and infection process on plant tissue were conducted to confirm the potential biological control value of the strain.

## Results

### Isolation and screening of strains antagonistic to *M*. *oryzae*

**Fig 1:**
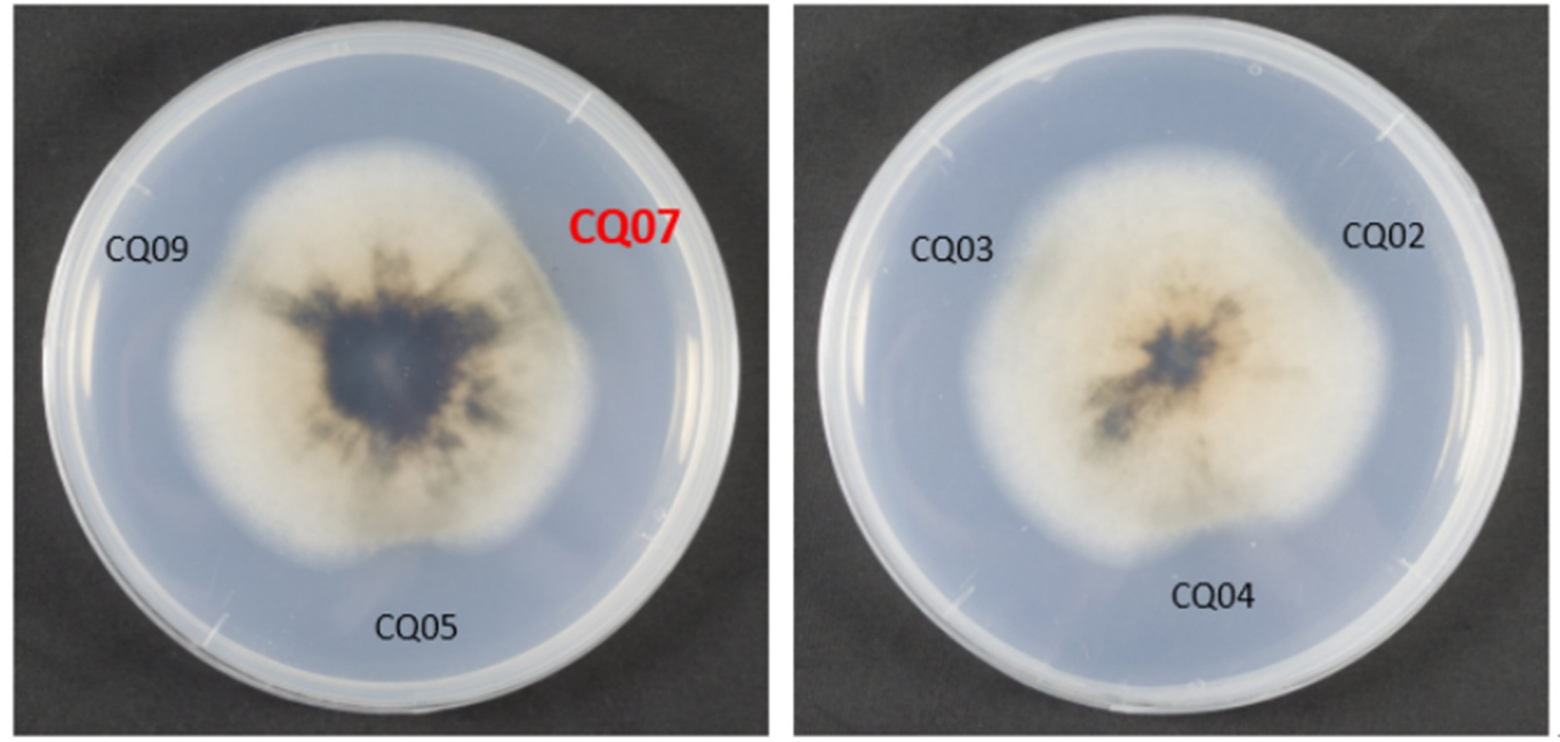
CQ07 showed a strong inhibitory effect against *M*. *oryzae* Guy11. CQ02, CQ03, CQ04, CQ05, CQ07, and CQ09 all showed inhibition against *M*. *oryzae* GUY11, but CQ07 had the strongest inhibition effect.

The soil was a mixture of soil from 800 to 3000 meters above sea level provided by the College of Resource Science and Technology of Sichuan Agricultural University. Twelve strains of bacteria antagonistic to the growth of *M*. *oryzae* were isolated from western Sichuan subalpine soil. Six sterilized culture filtrates of different colonies displayed obvious inhibitory effects against *M*. *oryzae* Guy11 by testing for mycelial growth inhibition. Among the antagonistic bacteria, the inhibition of CQ07 was particularly strong (Fig 1).

### Identification of strain CQ07

**Fig 2:**
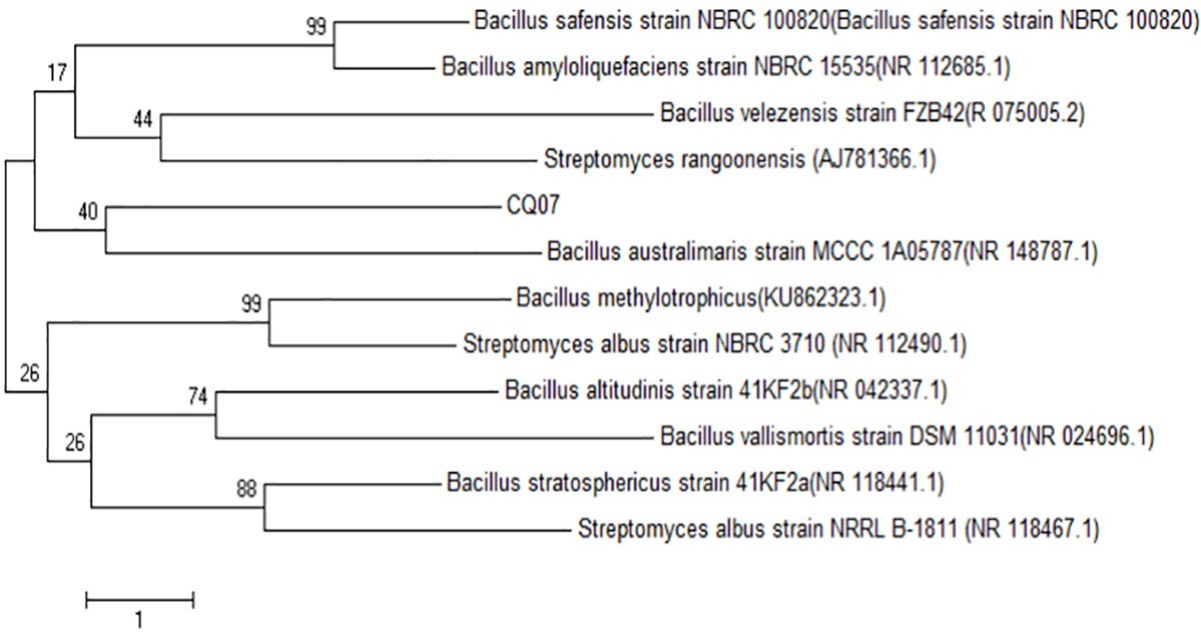
Neighbor-joining phylogenetic tree of CQ07 based on 16S rRNA sequence analysis.

**Fig 3:**
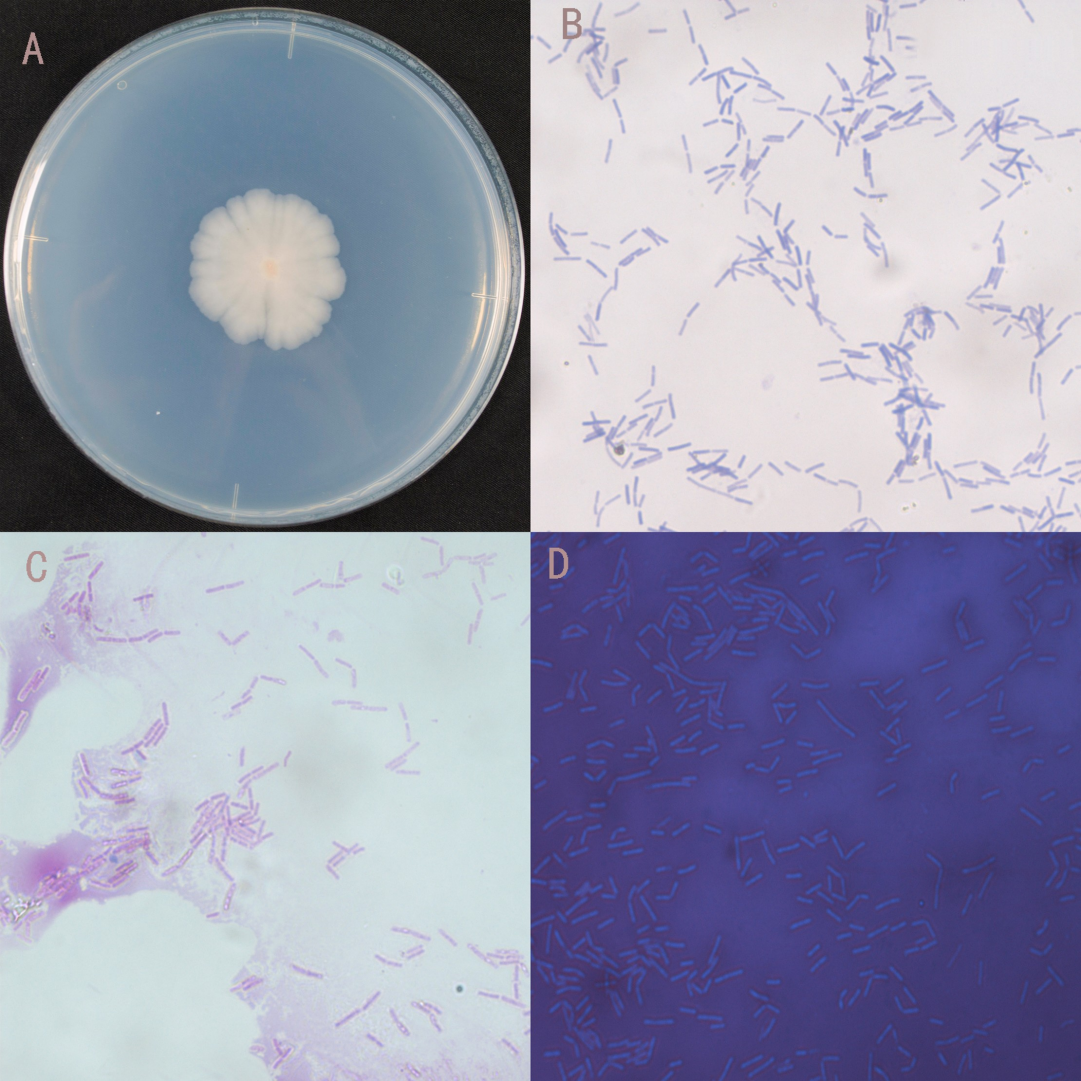
Morphological characteristics of CQ07. A. Colony morphology of CQ07. B. Gram staining of CQ07. C. Spore staining of CQ07. D. Capsule staining of CQ07.

16S rRNA sequence analysis indicated that strain CQ07 displayed approximately 99% similarity to *Bacillus australimaris*. A phylogenetic tree displaying the relationship between strain CQ07 and other strains is shown in Fig 2. The morphological and physiological characteristics of CQ07 are summarized in Fig 3 and Table 1. The results showed that CQ07 was a strain with an orange-red center and feathery edges. Gram and spore staining tests were positive; therefore, CQ07 was gram-positive and spore-producing. The capsule staining was negative, which suggests that CQ07 has no capsule.

**Table 1:** Physiological and biochemical results of strains CQ07.

Items	CQ07
Shape	Rod
Anaerobic growth	+
Motility	+
β-Galactoside	-
Utilization of lactose	-
Utilization of raffinose	-
Nitrate reduction to nitrite	+
Utilization of citrate	-
Mannitol	-
Indole test	-
L-Arabinose	-
Xylose	+
Oxidase activity	+
Decomposition of starch	+
Methyl red (MR) test	-
Voges-Proskauer (VP) tests	+
Growth in 7% NaCl	-
Growth in 8% NaCl	-
Growth in 9% NaCl	-

+: positive

-: negative

### Germination of conidia

**Fig 4:**
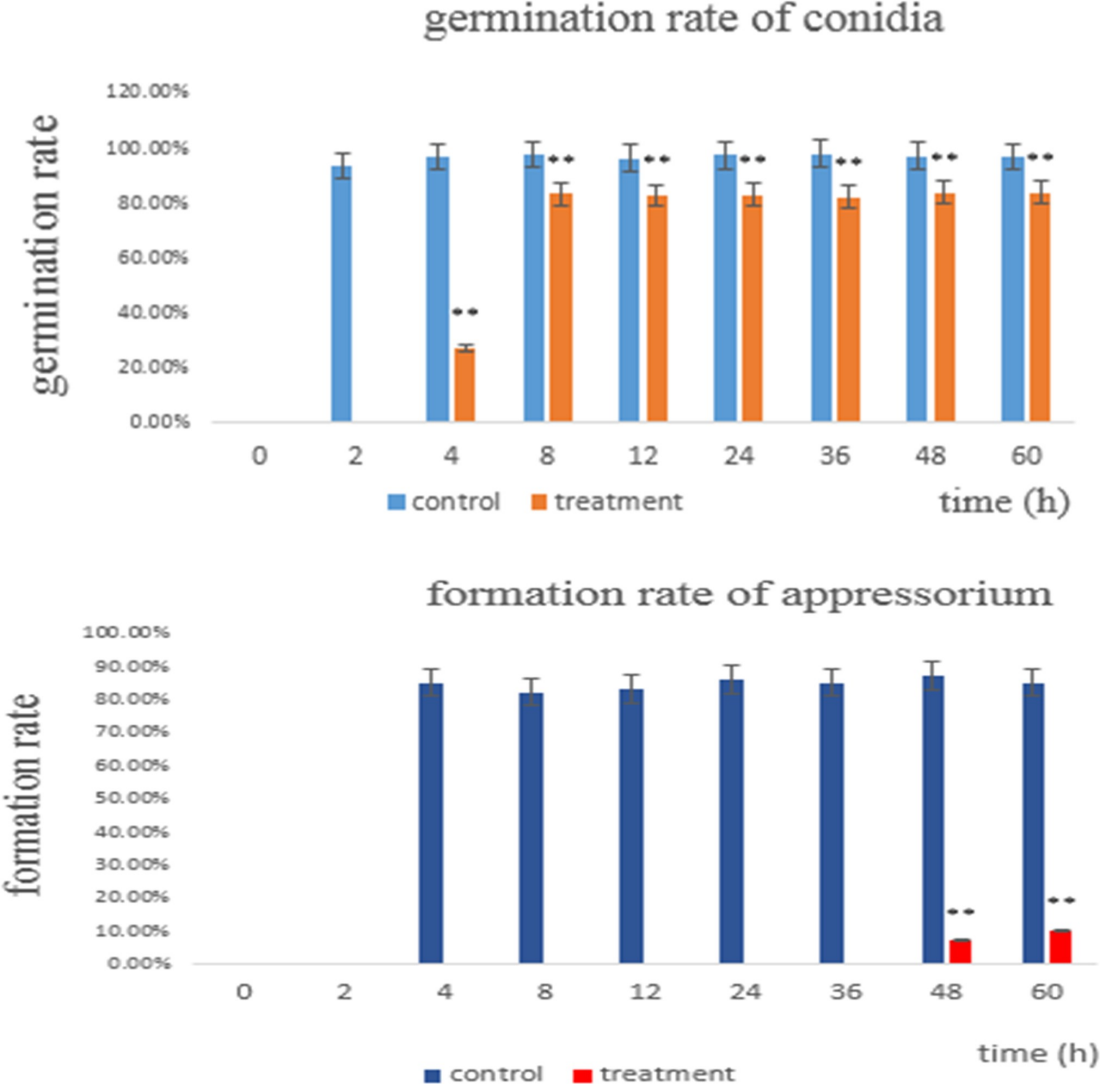
Germination rate of conidia at different times. The control group had already germinated after 2 hours, while the treatment group had not yet germinated. At 4 hours, the treatment group began to germinate, but the rate of germinated conidia was only 27%. After 8 hours, the germination rate of the treatment group was almost always stable at 83%, while the germination rate of the control group was 97%.

As shown in Fig 4, the germination rate of the control group reached 93% after 2 h, while the germination rate of the treatment was 0%. At 4 hours, the treatment group germination rate reached 27%, while the control group reached 96% and formed the appressorium. The germination rate after 8 hours of treatment reached 83% and was most stable in this range. However, the control group almost always achieved 97%. On the basis of these results, we can conclude that the culture filtrate of CQ07 can delay even suppress the germination of conidia.

### Formation of appressorium

**Fig 5:**
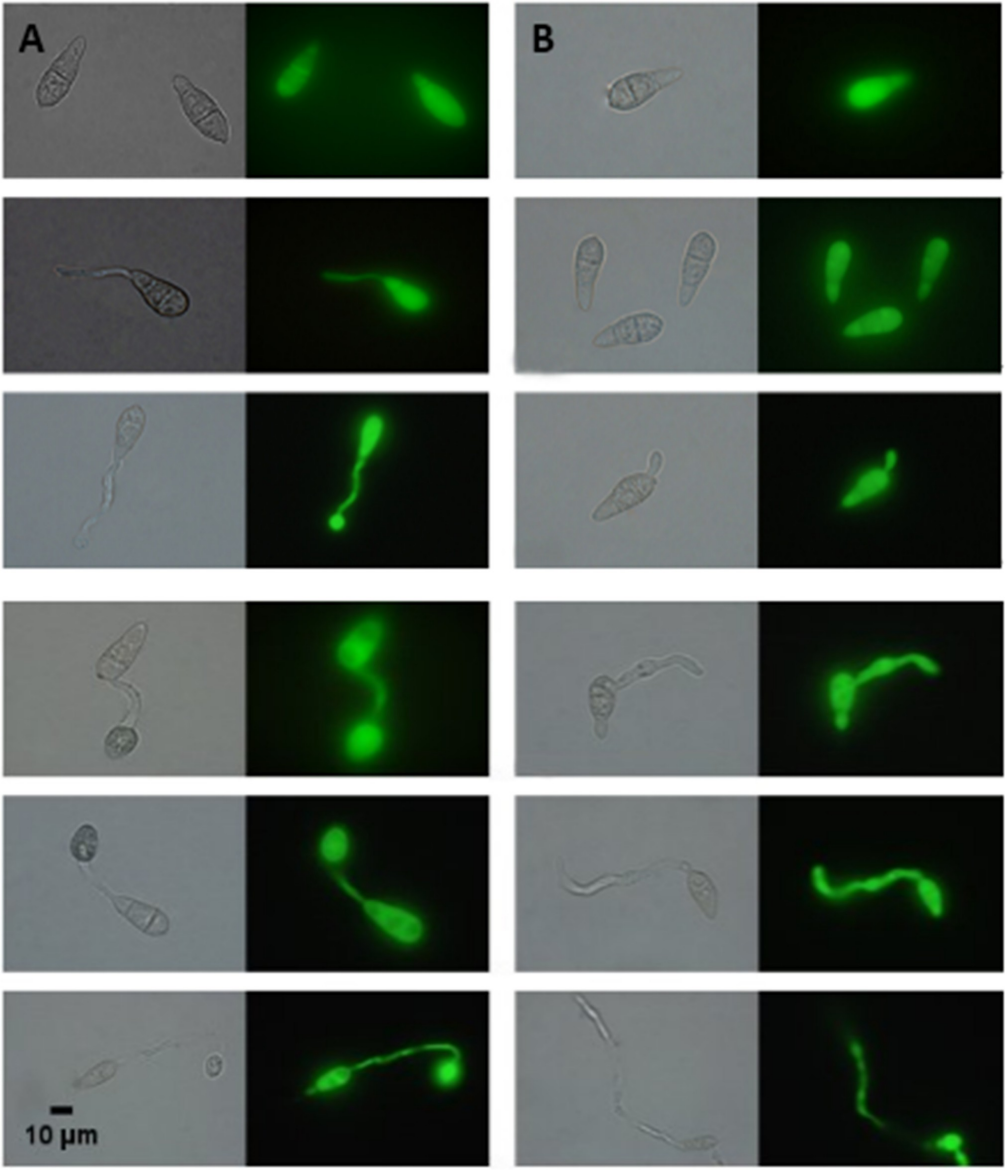
**A. Germination of conidia at different time points in the control group. B. Germination of conidia at different time points in the treatment group.** At 4 hours, 85% of appressorium of the control group was formed. After 8 hours, endlessly extended and unusual mycelium growth were observed in the treatment group. After 24 hours, the appressorium of the treatment group still did not form. However, after 48 hours, 7% of appressorium was observed in the treatment group. After 60 hours, the appressorium formation rate of the treatment group reached only 10%, while that of the control group reached 85%.

As shown in Fig 4 and Fig 5, the treatment group (Fig 5B) did not form an appressorium after 4 h, whereas the control group had an approximately 85% formed appressorium (Fig 5A). Even after 24 h, the appressorium was still not observed, except for the endlessly extended and unusual mycelium growth.

However, at 48 h and 60 h, 7% and 10% appressorium of the treatment, respectively, was found. This result also revealed why rice is still mildly infected when testing isolated leaves and living leaves and indicated that the sterilized culture filtrate of CQ07 delayed and inhibited the majority of appressorium formation, which is a necessary infection structure for the pathogenic ability of *M*. *oryzae*.

### The integrity of the cell membrane was destroyed by CQ07 culture filtrate

**Fig 6:**
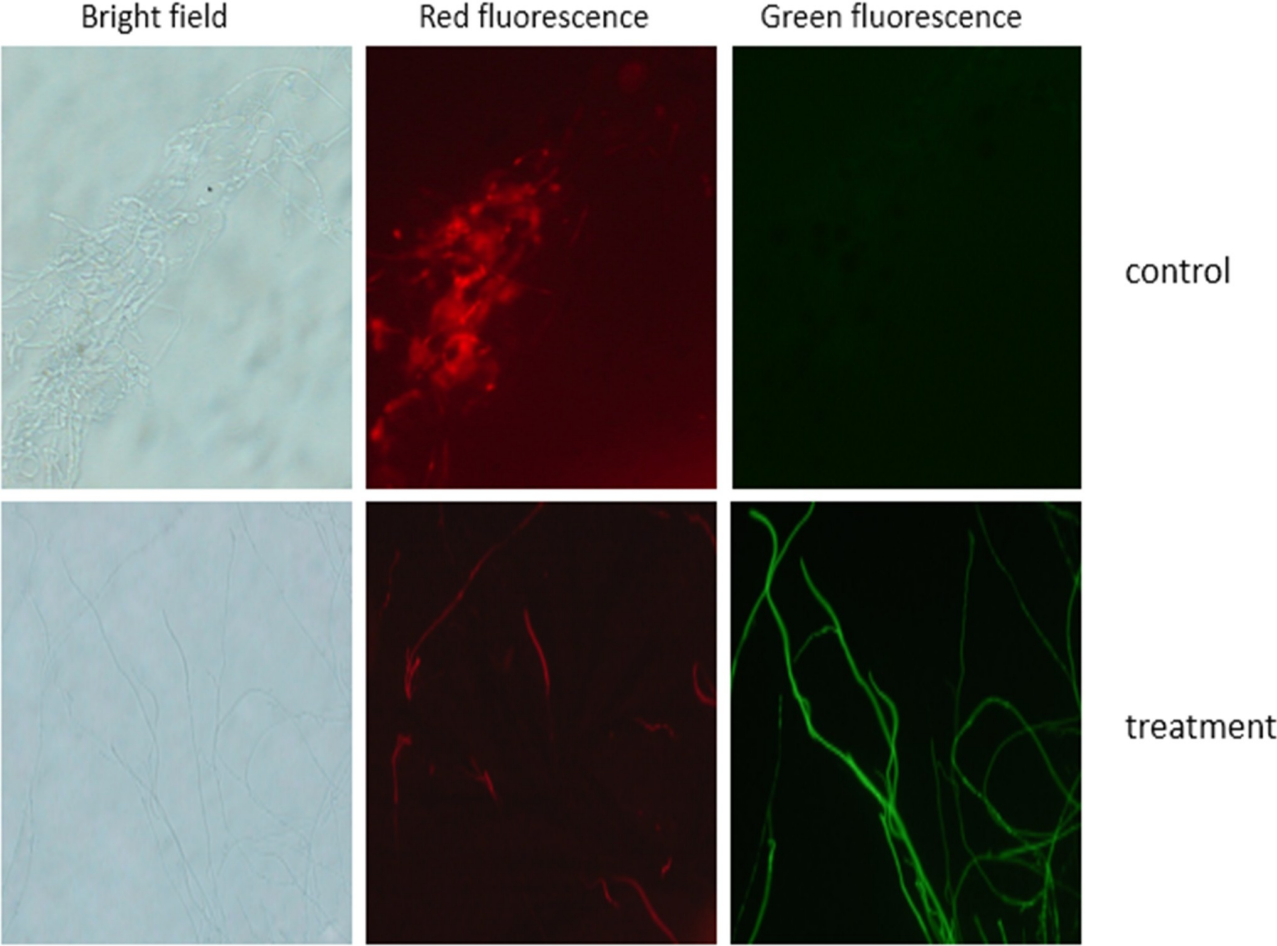
The cell membrane of the treatment group was destroyed by the culture filtrate of CQ07. Through the FDA-PI staining test, the mycelium of the control group showed the most green fluorescence. The mycelia of the treatment group all showed red fluorescence.

FDA and PI are two types of biological compound dyes that strain dead and alive cells, displaying different fluorescence through the cellular membrane permeability principle[14]. As shown in Fig 6, the cell membrane of *M*. *oryzae* was destroyed by CQ07 culture filtrate. Combining this test with the appressorium formation test, the mycelium displayed unusual growth in both these two tests. Furthermore, the cell membrane permeability test suggested that the permeability of swell parts was also destroyed.

### Observation of the infection process

**Fig 7:**
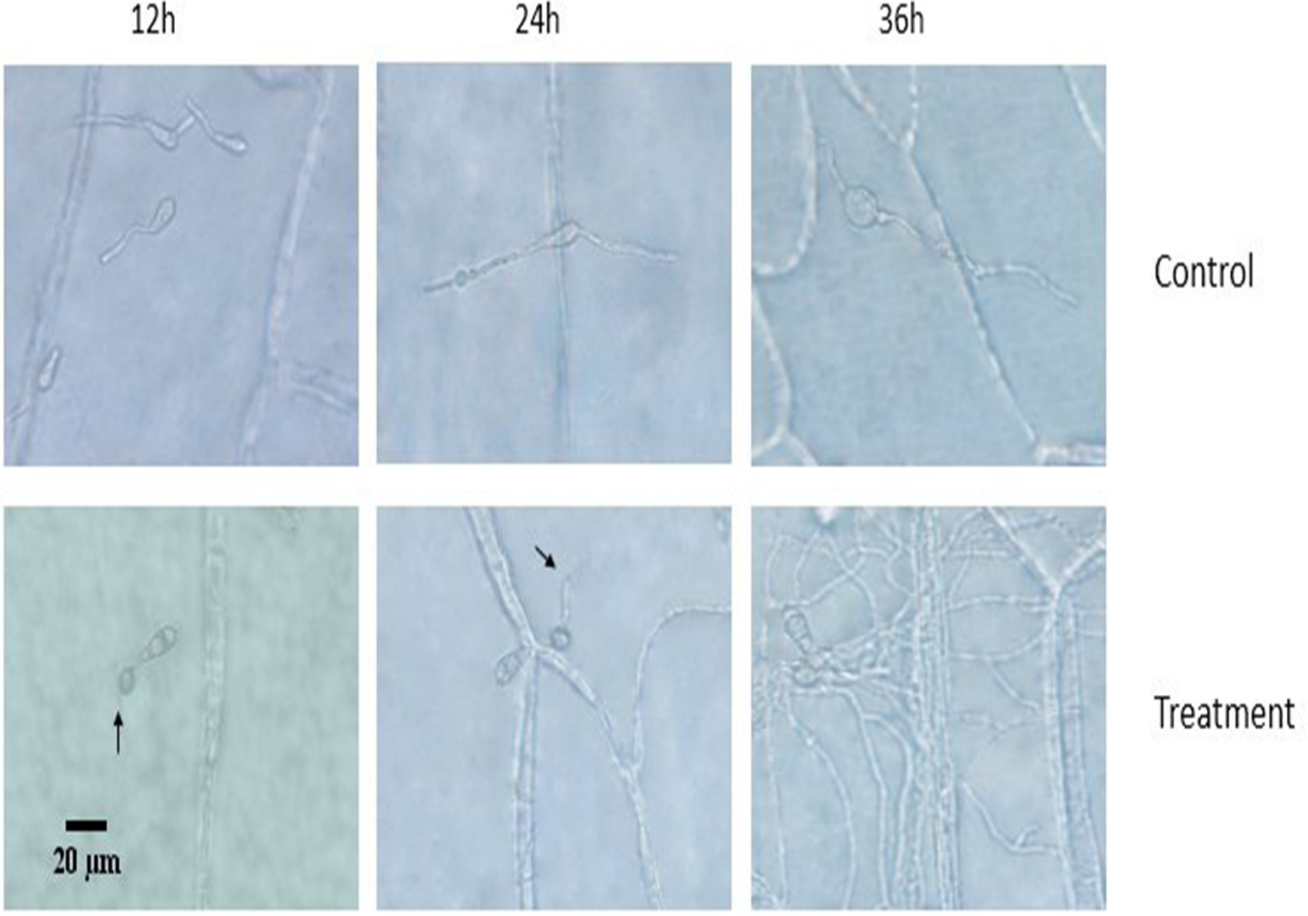
Infection status of conidia at different times. At 12 hours, appressorium of the control group was observed. At 24 hours, infection pegs of the control group were observed. At 36 hours, the mycelium of the control group infected the neighboring cells. At 24 and 36 hours, the treatment group only extended the malformed mycelium on the surface of the onion cells.

In agreement with germination and appressorium formation tests on hydrophobic slides in vitro, the conidia on onion epidermis did not germinate in the expected amount of time or form appressorium at the end of the growth period. As shown in Fig 7, the appressorium of the control group had already formed, and secondary mycelium extended into the onion cells at 24 h, whereas the mycelium infected the neighboring cells at 36 h. As shown in the treatment group shown in Fig 7, the treatment group only extended the malformed mycelium on the surface of the onion cells. From above, we can speculate that the filtrate of CQ07 still inhibits the activity of conidia in vivo.

### Evaluation of inhibition in vivo

**Fig 8:**
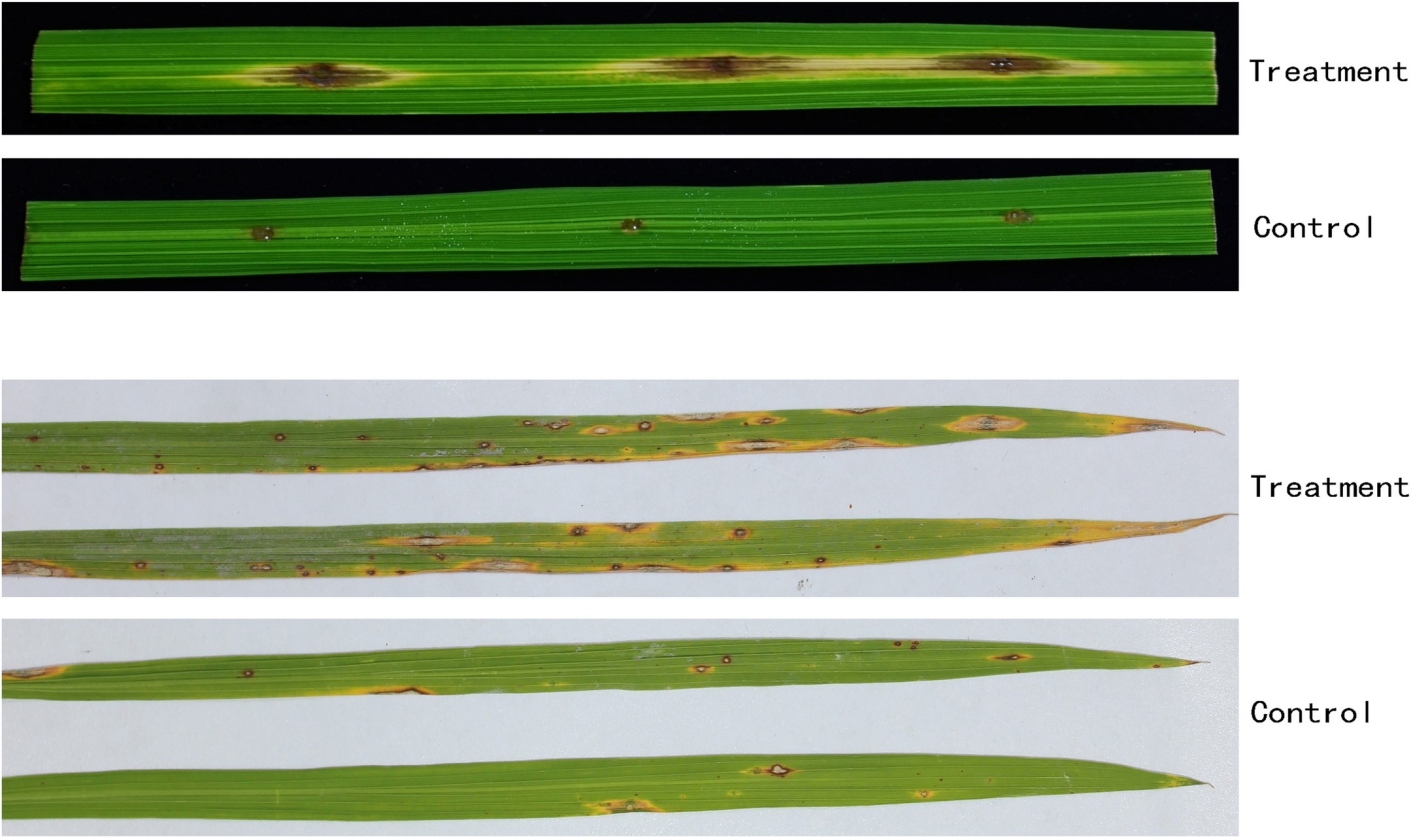
Pathogenicity of conidia on isolated and living leaves. The experiment on isolated leaves showed that the average lesion diameter of the control group was 10.3 mm, while the diameter of the treatment group was only 2.8 mm.The experiment on living leaves showed 24 lesions on average per leaf in the control group and 6 lesions on average per leaf in the treatment group.

The inhibitory pathogenicity of CQ07 against rice blasts is shown in Fig 8. In Fig 8, compared with the control group, the lesion diameter on isolated leaves of the treatment group was obviously smaller than that of the control group. The average lesion diameter of the control group in isolated leaves reached 10.3 mm, while the diameter of the treatment group was only 2.8 mm. The lesion quantity on living leaves of the control group was 24 lesions per leaf on average, while the lesion quantity of treatment was 6 lesions per leaf on average. This phenomenon indicated that the sterilized culture filtrate of CQ07 had a strong ability to inhibit the pathogenicity of *M*. *oryzae*, and the number of lesions decreased by 75% compared with that in the control group.

## Discussion

In nature, antagonism among different species is ubiquitous. For a long time, interest has focused on taking full advantage of antagonism, applying it to many fields such as agricultural defense or the medical domain. Plant fungal diseases are difficult to control, which leads to very large losses in economic crops. The leading strategy applied to control plant fungal disease is chemical pesticides, which cause serious environmental pollution[15]. Thus, strategies to protect plants from *M*. *oryzae* infection should be developed, especially environmentally friendly biological control methods.

Rice blasts have received considerable attention because of the increasing importance of food security worldwide, especially in East Asia. *M*. *oryzae* is one of the causal pathogenic fungi of rice blast disease. Host plants of *M*. *oryzae* are not only rice but also other Poaceae crops and their wild relatives, such as wheat, barley, finger millet, foxtail and wild grasses. The losses of rice harvest caused by rice blast reached 10–30% each year[16–18].

Many species of bacteria, especially Bacillus, are known as biological control agents that inhibit several phytopathogenic fungi due to the production of cell wall-degrading enzymes and other antifungal metabolic products[19–21]. Many Bacillus strains antagonistic to fungi have been isolated from soil[10, 22–24]. In our current study, several antagonism strains were isolated from western Sichuan subalpine soil. CQ07 was one of the most active strains that inhibited the growth of *M*. *oryzae*. The sterilized culture filtrate of CQ07 also significantly inhibited the growth of *M*. *oryzae* on nutrient medium. In the present study, based on the physiological and biochemical characteristics and 16S rRNA sequence analysis, CQ07 was identified as *B*. *australimaris*. To the best of our knowledge, this is the first isolation of *B*. *australimaris* from western Sichuan subalpine soil.

Germination of conidia and formation of appressorium are two crucial processes of *M*. *oryzae* during plant infection. However, through the results of the conidia germination test, the process of germination was obviously delayed and even completely suppressed. The conidia treated with sterilized culture filtrate did not germinate after 2 hours compared with those in the control group. After 4 hours, the control group had already formed a dome-shaped appressorium, while the treated group reached a germination rate of only 27% and exhibited a short germ tube. At 8 hours and afterwards, the germ tube of the treated group began to extend without limits, and unusual growths emerged on the mycelium. Only after 48 hours, there was 7% appressorium began to form. The results shown above definitely illustrate that the germination of conidia was delayed and inhibited and the formation of appressorium was hindered by the crude fermentation filtrate of CQ07. Therefore, we can deduce that the filtrate of CQ07 can inhibit the formation of infectious structures of *M*. *oryzae* in vitro.

Cellular activity is observed by cell permeability, and the permeability of *M*. *oryzae* was destroyed by the culture filtrate of CQ07 through the FDA-PI dyeing experiment. Dynamic observation of the infection process and pathogenicity test in vivo can verify the impact of the CQ07 filtrate against the pathogenicity of *M*. *oryzae*. Onion epidermis is an ideal material to observe the infection process because of its large cells, easy access and convenience to observe. Researchers have already utilized onion epidermis to examine the infection of *M*. *oryzae*[25, 26]. Therefore, we used onion epidermis to perform pathogenicity test observations. Through the test results, we observed that the conidia handled with the culture filtrate of CQ07 lost pathogenicity. From the image, we can see that the appressorium and infection peg were only observed in the control group at 12 h and 24 h. Only long and unusual mycelia were observed on the surface of onion cells in the treatment group.

As shown in Fig, the lesion of the control group was obviously larger than that of the treatment group. Furthermore, this result was consistent with the phenomenon in the living leaf experiment. The number of lesions in the treatment group was much lower than that in the control group. By evaluating the infection situation, we found that the pathogenicity of *M*. *oryzae* was significantly inhibited.

From the above, we can deduce that CQ07 can inhibit not only the growth, germination, and formation of appressorium in vitro but also the pathogenicity of *M*. *oryzae* in vivo. Therefore, we can conclude that CQ07 might effectively inhibit infection by influencing the formation of the infectious structure of *M*. *oryzae*. However, the formation of infectious structures is regulated by various genes, and the process is complicated. In future studies, we will explore the molecular mechanism related to our present studies.

In summary, *B*. *australimaris* CQ07 is not only a candidate biological control agent, but its ability to inhibit pathogenicity has furthered the progress of pathogenesis research.

## Methods

### Isolation of antagonistic strains from soil

The western Sichuan subalpine soil used to isolate the bacteria was provided by the College of Resource Science and Technology of Sichuan Agricultural University. Then, 1 g of soil was suspended in 10 ml of sterilized water and vortexed for 1 min before centrifugation at 2000 rpm for 10 min. Then, 0.1 mL of supernatant was diluted and plated onto potato dextrose agar[27]. After cultivation at 37°C for 48 h in a homothermal incubator, strains that appeared on nutrient agar plates were isolated as single colonies on potato dextrose medium.

### Screening of the antagonistic bacteria against *M*. *oryzae* in vitro

The rice blast pathogenic fungus *M*. *oryzae* Guy11 was provided by the Plant Pathogenic Laboratory of Sichuan Agricultural University. To select the antagonistic strains, every strain was inoculated in homologous fluid nutrient medium and incubated at 28°C for 48 h in a rotary shaker at 180 rpm. One milliliter of sterilized culture filtrate from every strain was removed and stored in a small EP tube. Ten microliters of sterilized culture filtrate of each strain was removed from every small EP tube and injected into the edge of a *M*. *oryzae* colony, which had been deposited in the center of potato dextrose agar medium. After the sterilized culture filtrate of the antagonistic strain was injected, the *M*. *oryzae* medium was incubated at 28°C in an incubator. After 2 or 3 days, the inhibition of growth of *M*. *oryzae* was compared by observing the degree of diminished growth. The strongest antagonistic bacteria were named CQ07 and taken for further study.

### Morphological characteristics and 16S rRNA analysis of CQ07

The method of extracting total genomic DNA was based on the manufacturer’s instructions of a commercial DNA extraction kit. The 16S rRNA of the strain was amplified using forward and reverse primers in a small EP tube. The polymerase chain reaction program proceeded in a PCR thermocycle instrument. The PCR products were purified using a PCR Purification Kit, and the PCR products were identified by horizontal electrophoresis on a 1% agarose gel. The fragments of amplified 16S rRNA were transformed into *Escherichia coli*. Sequences were sequenced by TSINGKE (Chengdu, China). The similarity of 16S rRNA sequences was compared using the BLAST search program in the NCBI database (http://www.ncbi.nlm.nih.gov).

A colony of CQ07 was observed on PDA medium. Bacteriological, biochemical and physiological properties were tested according to Bergey’s Manual.

### Germination test of conidia

The sterilized culture filtrate and sterilized water were used to scrape the conidia from 10-day-old *M*. *oryzae* conidia-producing medium, which was adjusted to a concentration of 1×10^5^ conidia/ml after filtration with two layers of lens paper[28]. Fifty microliters of conidia suspension was dropped on the hydrophobic cover slide. Then, the hydrophobic cover slides were placed in a moist petri dish to germinate at room temperature. After 0, 2, 4, 8, 12, 24, 36, 48, and 60 hours, the germination degree of conidia was observed under a ZEISS microscope, and 100 conidia were selected to calculate the germination rate. The experiment was repeated three times.

The formula for the germination rate was as follows:

Germination rate (%) = [R1/R2] ×100%

where R1 = the number of germinated conidia and

R2 = the number of total conidia.

### Formation of appressorium

Conidia were scraped with sterilized culture filtrate and sterilized water from 10-day-old *M*. *oryzae* conidia-producing medium, then the conidia suspension was diluted to 1×10^5^ conidia/ml. Fifty microliters of conidia per droplet were dripped on a hydrophobic cover slide. After incubation at room temperature in a moist petri dish for 0, 2, 4, 8, 12, 24, 36, 48, and 60 hours, appressorium formation was observed under a ZEISS fluorescence microscope. The experiment was repeated three times.

The formula for appressorium formation rate was as follows:

Formation rate of appressorium(%) = [X_1_/X_2_] ×100%

X_1_: the number of conidia that formed appressorium;

X_2_: the number of total conidia.

### Experiment of cell activity

*M*. *oryzae* was cultivated with 99 ml of fluid medium at 28°C for 24 h, and then 1 ml of sterilized culture filtrate was added to the fluid medium. After the *M*. *oryzae* continued cultivation in the mixed medium for 24 h, the mycelia of *M*. *oryzae* were cut off and dyed with FDA-PI composite dyes[14] for 5~10 min under dark conditions before being observed under a ZEISS fluorescence microscope at 20 times magnification. The experiment was repeated three times.

### Experiment of the infection process

The isolated strain that demonstrated the greatest suppression activity against *M*. *oryzae* in vitro was observed with respect to its ability to inhibit rice blast in plant tissue. The onion epidermis was divided into two groups, and each group had 3 epidermis samples. The epidermis was injected with a 50 μl of conidia suspension (concentration of 1×10^5^ conidia/ml) that was treated with two kinds of liquid as detailed below: sterilized water and sterilized culture filtrate of CQ07. The infection status was observed under a ZEISS microscope at the corresponding time (12, 24, and 36 h). The experiment was repeated three times.

### Experiment on isolated leaves

Rice seedling were grown at artificial climate chamber (30°C) for 30 days. Leaves of rice without obvious symptoms of disease were placed in petri dishes with a solution of 6-benzylaminopurine. Prior to that, every leaf was poked slightly with a needle, which made leaves more susceptible to *M*. *oryzae* infection.

Treatment group: a 5 μL volume of *M*. *oryzae* conidia suspension (1 × 10^5^ conidia/mL) droplets was diluted with a sterilized culture filtrate of CQ07 and applied to the slightly punctured sites of the leaves. Then, the leaves were incubated at 25°C in the dark for 24 h. Subsequently, all leaves were incubated in the light at 25°C[29]. In the control group, sterile water was used instead of the sterile culture filtrate, and other steps were unchanged. After 5 days, lesion length was compared.

The experiment was repeated three times.

### Experiment on living leaves

Considering that experiments in the isolated leaves do not accurately reflect inhibitory abilities under field conditions, we carried out simulate field tests aiming to confirm the biological control effects of CQ07. The experiment was finished under growth climate chamber conditions. The bioassay was conducted in accordance with previous methods[30]. First, 30-day-old rice seedlings were transferred to an inoculation chamber(30°C). In the treatment experiment, the rice seedlings were sprayed to runoff with the conidia suspension of *M*. *oryzae* (1× 10^5^ conidia/mL) diluted by sterilized culture filtrate of CQ07 containing Tween 20. The control group was treated in the same way, but the sterile water was in place of the culture filtrate. After 5 days, lesion quantity was compared (both 100 leaves of control group and treatment group were counted; the experiment was repeated three times).

The formula for the inhibition rate was as follows:

Inhibition rate (%) = [N_1_-N2_2_/ N_1_] ×100%

N_1_: lesion quantity before treatment;

N_2_: lesion quantity after treatment

## Supporting information

S1 Data Raw data for Fig 4. (XLSX)

S2 Data Raw data of Fig 8. (XLSX)

S1 File 16S rRNA sequence of CQ07. (DOCX)

## References

[1] Zhang H, Wu Z, Wang C, Li Y, Xu JR. Germination and infectivity of microconidia in the rice blast fungus Magnaporthe oryzae. Nature Communications. 2014;5:4518 10.1038/ncomms5518 25082370 PMC4143928

[2] Xiong ZQ, Tu XR, Wei SJ, Huang L, Li XH, Lu H, et al In vitro antifungal activity of antifungalmycin 702, a new polyene macrolide antibiotic, against the rice blast fungus Magnaporthe grisea. Biotechnology Letters. 2013;35(9):1475 10.1007/s10529-013-1229-z 23690041

[3] Li Q, Jiang Y, Ning P, Zheng L, Huang J, Li G, et al Suppression of Magnaporthe oryzae by culture filtrates of Streptomyces globisporus JK-1. Biological Control. 2011;58(2):139–48.

[4] Ohtaka N, Kawamata H, Narisawa K. Suppression of rice blast using freeze-killed mycelia of biocontrol fungal candidate MKP5111B. Journal of General Plant Pathology. 2008;74(2):101–8.

[5] Hyakumachi M, Takahashi H, Matsubara Y, Someya N, Shimizu M, Kobayashi K, et al Recent studies on biological control of plant diseases in Japan. Journal of General Plant Pathology. 2014;80(4):287–302.

[6] Vidhyasekaran P, Rabindran R, Muthamilan M, Nayar K, Rajappan K, Subramanian N, et al Development of a powder formulation of Pseudomonas fluorescens for control of rice blast. Plant Pathology. 2010;46(3):291–7.

[7] Sha Y, Wang Q, Li Y. Suppression of Magnaporthe oryzae and interaction between Bacillus subtilis and rice plants in the control of rice blast. Springerplus. 2016;5(1):1238 10.1186/s40064-016-2858-1 27536521 PMC4971003

[8] Karthikeyan V, Gnanamanickam SS. Biological control of Setaria blast (Magnaporthe grisea) with bacterial strains. Crop Protection. 2008;27(2):263–7.

[9] Mu C, Liu X, Lu Q, Jiang X, Zhu C. Biological control of rice bmast by Bacillus subtilis B-332 strain. Acta Phytophylacica Sinica. 2007:123–8.

[10] Shan H, Zhao M, Chen D, Cheng J, Li J, Feng Z, et al Biocontrol of rice blast by the phenaminomethylacetic acid producer of Bacillus methylotrophicus strain BC79. Crop Protection. 2013;44(1):29–37.

[11] Zhao ZZ, Wang QS, Wang KM, Brian K, Liu CH, Gu YC. Study of the antifungal activity of Bacillus vallismortis ZZ185 in vitro and identification of its antifungal components. Bioresour Technol. 2010;101(1):292–7. 10.1016/j.biortech.2009.07.071 19717300

[12] Ebbole DJ. Magnaporthe as a model for understanding host-pathogen interactions. Annual Review of Phytopathology. 2007;45(1):437–56. 10.1146/annurev.phyto.45.062806.094346 17489691

[13] Howard RJ, Ferrari MA, Roach DH, Money NP. Penetration of hard substrates by a fungus employing enormous turgor pressures. Proceedings of the National Academy of Sciences of the United States of America. 1991;88(24):11281–4. 10.1073/pnas.88.24.11281 1837147 PMC53118

[14] Xiao X, Han ZY, Chen YX, Liang XQ, Li H, Qian YC. Optimization of FDA–PI method using flow cytometry to measure metabolic activity of the cyanobacteria, Microcystis aeruginosa. Physics & Chemistry of the Earth. 2011;36(9):424–9.

[15] Raaijmakers JM, Vlami M, Souza JTD. Antibiotic production by bacterial biocontrol agents. Antonie Van Leeuwenhoek. 2002;81(1–4):537–47. 12448749 10.1023/a:1020501420831

[16] Yoshida K, Saunders DGO, Mitsuoka C, Natsume S, Kosugi S, Saitoh H, et al Host specialization of the blast fungus Magnaporthe oryzae is associated with dynamic gain and loss of genes linked to transposable elements. BMC Genomics. 2016;17(1):1–18. 27194050 10.1186/s12864-016-2690-6 PMC4870811

[17] Kato H, Yamamoto M, Yamaguchi-Ozaki T, Kadouchi H, Iwamoto Y, Nakayashiki H, et al Pathogenicity, Mating Ability and DNA Restriction Fragment Length Polymorphisms of Pyricularia Populations Isolated from Gramineae, Bambusideae and Zingiberaceae Plants. Journal of General Plant Pathology. 2000;66(1):30–47.

[18] Talbot NJ. On the Trail of a Cereal Killer: Exploring the Biology of Magnaporthe grisea. Annual Review of Microbiology. 2003;57(1):177–202. 10.1146/annurev.micro.57.030502.090957 14527276

[19] Sellem I, Triki MA, Elleuch L, Cheffi M, Chakchouk A, Smaoui S, et al The use of newly isolated Streptomyces strain TN258 as potential biocontrol agent of potato tubers leak caused by Pythium ultimum. Journal of Basic Microbiology. 2017;57(5):393 10.1002/jobm.201600604 28217886

[20] Han T, You C, Zhang L, Feng C, Zhang C, Wang J, et al Biocontrol potential of antagonist Bacillus subtilis Tpb55 against tobacco black shank. Biocontrol. 2016;61(2):1–11.

[21] Calvo H, Marco P, Blanco D, Oria R, Venturini ME. Potential of a new strain of Bacillus amyloliquefaciens BUZ-14 as a biocontrol agent of postharvest fruit diseases. Food Microbiology. 2017;63:101–10. 10.1016/j.fm.2016.11.004 28040156

[22] Yilmaz M, Soran H, Beyatli Y. Antimicrobial activities of some Bacillus spp. strains isolated from the soil. Microbiological Research. 2006;161(2):127–31. 10.1016/j.micres.2005.07.001 16427515

[23] Gao M, Li R, Dai S, Wu Y, Yi D. Diversity of Bacillus thuringiensis strains from soil in China and their pesticidal activities. Biological Control. 2008;44(3):380–8.

[24] Chaves JQ, Pires ES, Vivoni AM. Genetic diversity, antimicrobial resistance and toxigenic profiles of Bacillus cereus isolated from food in Brazil over three decades. International Journal of Food Microbiology. 2011;147(1):12–6. 10.1016/j.ijfoodmicro.2011.02.029 21440319

[25] Géraldine M, Katrin H, Jan S, Tenberge KB, Paul T. CPMK2, an SLT2-homologous mitogen-activated protein (MAP) kinase, is essential for pathogenesis of Claviceps purpurea on rye: evidence for a second conserved pathogenesis-related MAP kinase cascade in phytopathogenic fungi. Molecular Microbiology. 2010;46(2):305–18. 10.1046/j.1365-2958.2002.03133.x 12406210

[26] Wang J, Du Y, Zhang H, Zhou C, Qi Z, Zheng X, et al The actin-regulating kinase homologue MoArk1 plays a pleiotropic function in Magnaporthe oryzae. Molecular Plant Pathology. 2013;14(5):470–82. 10.1111/mpp.12020 23384308 PMC3642230

[27] Sevgi E, Coral G, GiZiR AM, Sangün MK. Investigation of heavy metal resistance in some bacterial strains isolated from industrial soils. Turkish Journal of Biology. 2010;34(4):423–31.

[28] Zhang H, Zhao Q, Liu K, Zhang Z, Wang Y, Zheng X. MgCRZ1, a transcription factor of Magnaporthe grisea, controls growth, development and is involved in full virulence. Fems Microbiology Letters. 2010;293(2):160–9. 10.1111/j.1574-6968.2009.01524.x 19260966

[29] Park CH, Shirsekar G, Bellizzi M, Chen S, Songkumarn P, Xie X, et al The E3 Ligase APIP10 Connects the Effector AvrPiz-t to the NLR Receptor Piz-t in Rice. Plos Pathogens. 2016;12(3):e1005529 10.1371/journal.ppat.1005529 27031246 PMC4816579

[30] Ying-Lao Z, Shuai L, Dong-Hua J, Li-Chun K, Ping-Hua Z, Jia-Dong X. Antifungal activities of metabolites produced by a termite-associated Streptomyces canus BYB02. Journal of Agricultural & Food Chemistry. 2013;61(7):1521–4. 23360202 10.1021/jf305210u

