# Supplementary material for: The isolation of the antagonistic strain Bacillus australimaris CQ07 and the exploration of the pathogenic inhibition mechanism of Magnaporthe oryzae
Source: PLoS One. 2019 Aug 12;14(8):e0220410. doi: 10.1371/journal.pone.0220410 (PMC6690535; doi:10.1371/journal.pone.0220410)
Supplement: S1 File — (DOCX) [file pone.0220410.s003.docx]

**16S rRNA base sequence of CQ07 (1577bp):**

GATTCGCGTGTCGCCCTTCAGAGTTTGATCCTGGCTCAGGACGAACGCTG

GCGGCGTGCCTAATACATGCAAGTCGAGCGGACAGAAGGGAGCTTGCTCC

CGGATGTTAGCGGCGGACGGGTGAGTAACACGTGGGTAACCTGCCTGTAA

GACTGGGATAACTCCGGGAAACCGGAGCTAATACCGGATAGTTCCTTGAA

CCGCATGGTTCAAGGATGAAAGACGGTTTCGGCTGTCACTTACAGATGGA

CCCGCGGCGCATTAGCTAGTTGGTGGGGTAATGGCTCACCAAGGCGACGA

TGCGTAGCCGACCTGAGAGGGTGATCGGCCACACTGGGACTGAGACACGG

CCCAGACTCCTACGGGAGGCAGCAGTAGGGAATCTTCCGCAATGGACGAA

AGTCTGACGGAGCAACGCCGCGTGAGTGATGAAGGTTTTCGGATCGTAAA

GCTCTGTTGTTAGGGAAGAACAAGTGCGAGAGTAACTGCTCGCACCTTGA

CGGTACCTAACCAGAAAGCCACGGCTAACTACGTGCCAGCAGCCGCGGTA

ATACGTAGGTGGCAAGCGTTGTCCGGAATTATTGGGCGTAAAGGGCTCGC

AGGCGGTTTCTTAAGTCTGATGTGAAAGCCCCCGGCTCAACCGGGGAGGG

TCATTGGAAACTGGGAAACTTGAGTGCAGAAGAGGAGAGTGGAATTCCAC

GTGTAGCGGTGAAATGCGTAGAGATGTGGAGGAACACCAGTGGCGAAGGC

GACTCTCTGGTCTGTAACTGACGCTGAGGAGCGAAAGCGTGGGGAGCGAA

CAGGATTAGATACCCTGGTAGTCCACGCCGTAAACGATGAGTGCTAAGTG

TTAGGGGGTTTCCGCCCCTTAGTGCTGCAGCTAACGCATTAAGCACTCCG

CCTGGGGAGTACGGTCGCAAGACTGAAACTCAAAGGAATTGACGGGGGCC

CGCACAAGCGGTGGAGCATGTGGTTTAATTCGAAGCAACGCGAAGAACCT

TACCAGGTCTTGACATCCTCTGACAACCCTAGAGATAGGGCTTTCCCTTC

GGGGACAGAGTGACAGGTGGTGCATGGTTGTCGTCAGCTCGTGTCGTGAG

ATGTTGGGTTAAGTCCCGCAACGAGCGCAACCCTTGATCTTAGTTGCCAG

CATTTAGTTGGGCACTCTAAGGTGACTGCCGGTGACAAACCGGAGGAAGG

TGGGGATGACGTCAAATCATCATGCCCCTTATGACCTGGGCTACACACGT

GCTACAATGGACAGAACAAAGGGCTGCAAGACCGCAAGGTTTAGCCAATC

CCATAAATCTGTTCTCAGTTCGGATCGCAGTCTGCAACTCGACTGCGTGA

AGCTGGAATCGCTAGTAATCGCGGATCAGCATGCCGCGGTGAATACGTTC

CCGGGCCTTGTACACACCGCCCGTCACACCACGAGAGTTTGCAACACCCG

AAGTCGGTGAGGTAACCTTTATGGAGCCAGCCGCCGAAGGTGGGGCAGAT

GATTGGGGTGAAGTCGTAACAAGGTAGCCGTATCGGAAGGTGCGGCTGGA

TCACCTCCTAAGGGCGACACGCGAATT
